# SS31 Ameliorates Oxidative Stress *via* the Restoration of Autophagic Flux to Protect Aged Mice From Hind Limb Ischemia

**DOI:** 10.3389/fcvm.2022.789331

**Published:** 2022-04-14

**Authors:** Qiaoyun Yang, Chunqiu Li, Qingwei Chen

**Affiliations:** Department of General Practice, The Second Affiliated Hospital of Chongqing Medical University, Chongqing, China

**Keywords:** peripheral artery disease (PAD), Szeto-Schiller peptide, autophagic flux, oxidative stress, AKT-mTOR pathway

## Abstract

**Background:**

Oxidative stress and impaired autophagic flux play important roles in the development of peripheral artery disease (PAD). SS31 is considered an important antioxidant peptide and autophagy regulator. We aimed to investigate the role of SS31 in PAD myopathy and its possible mechanism both *in vivo* and *in vitro*.

**Methods:**

A hind limb ischemia (HLI) model was established with old C57BL/6 (14-month-old) mice. Mice in the SS31 group were intraperitoneally injected with SS31 (3 mg/kg) for 4 weeks. We examined skeletal muscle function and histomorphology, autophagy-related protein levels and reactive oxygen species (ROS) content. For the *in vitro* experiments, after C2C12 myotubes were treated with CoCl_2_, SS31, and chloroquine (CQ) or rapamycin (RAPA), we measured ROS content, autophagy-related protein levels and antioxidant enzyme expression.

**Results:**

SS31 treatment effectively enhanced the recovery of skeletal muscle function, alleviated skeletal muscle injury and suppressed mitochondrial ROS production in ischemic limbs. SS31 reduced apoptosis and oxidative stress, and SS31 restored impaired autophagic flux by inhibiting the AKT-mTOR pathway. *In vitro* studies showed that SS31 restored autophagic flux and improved oxidative stress in C2C12 cells. Moreover, phosphorylated AKT (p-AKT) and phosphorylated mTOR (p-mTOR) levels were reduced.

**Conclusion:**

These experiments indicated that SS31 can inhibit oxidative stress by restoring autophagic flux to reverse hypoxia-induced injury *in vivo* and *in vitro*.

## Introduction

Peripheral artery disease (PAD) is usually considered a lower extremity artery disease caused by arterial obstruction. The most common cause of PAD is atherosclerosis (1, 2). The most serious manifestation of PAD is critical limb ischemia (3). The skeletal muscle of PAD patients shows obvious myopathy, which is characterized by abnormalities in skeletal muscle function and morphology as well as metabolic defects (4, 5). The development of PAD is closely correlated with oxidative stress (6). The interconnection between reactive oxygen species (ROS) and oxidative stress leads to progressive dysfunction of the antioxidant defense system, which is closely related to PAD prognosis (7, 8). It has also been shown that apoptosis in hind limb ischemic mice is increased (9). Current therapies for PAD include lifestyle modifications, structured exercise therapy, pharmacotherapy and revascularization (10). PAD affects more than 200 million people globally, particularly people over 65 years old (11).

Autophagy is an essential and evolutionarily conserved cellular recycling process. Autophagic flux maintains the balance of the internal environment of cells, tissues and organisms by degrading damaged organelles and misfolded proteins and reusing the generated pool of biomolecules (12). Autophagic flux plays a “double-edged sword” role in cardiovascular disease. On the one hand, autophagy maintains normal cardiovascular function by degrading abnormal components in cells. On the other hand, excessive activation of autophagy underlies autophagic cell death and loss of cells (13–15). A study reported blockage of autophagic flux in skeletal muscle of critical limb ischemia, suggesting that autophagy is probably engaged in the occurrence and development of PAD (16). Therefore, we propose that restoration of impaired autophagic flux may be a promising approach to alleviating ischemic skeletal muscle injury.

The Szeto-Schiller peptide (SS-31) is a cell-permeable antioxidant peptide that is highly and selectively concentrated in the mitochondrial inner membrane and that confers mitochondrial protection by suppressing mitochondrial ROS production and inhibiting mitochondrial permeability transition (17–20). The protective effects of SS31 have been reported in several diseases, such as ischemic brain injury (21), acute kidney injury (AKI) (22), diabetes mellitus (23) and myocardial ischemia-reperfusion (IR) injury (24). SS31 has been shown to prevent IR injury by reducing mitochondrial dysfunction and oxidative stress (24). Surprisingly, the therapeutic role of peptide SS31 in PAD has been rarely reported. Therefore, in this study, we investigated the therapeutic potential of SS31 in the treatment of hind limb ischemia (HLI) and its mechanism of action.

## Materials and Methods

### Hind Limb Ischemia and Dosage Regimen

Forty C57BL/6 mice (male, 27–33 g, 14 months old) were purchased from the Animal Center of Chongqing Medical University and randomly assigned to two groups [the normal saline (NS) group and SS31 group]. After anesthesia with intraperitoneal injection of 2% pentobarbital (45 mg/kg), the mice were placed flat on a plastic sheet with tape. A single researcher revealed the left femoral artery and dissected the left femoral vein and nerve. Ligation and excision were performed on the proximal and distal ends of the left femoral artery of the mice in the HLI group. The ratio of blood perfusion between the operative side and the control side was recorded, and the model was considered to be successfully established when the reduction in blood flow on the operative side was ≥70% (comparison with blood flow on the control side). The blood flow was evaluated by moorLDI2-HIR (Moor Instruments, United Kingdom). The right limbs of the mice were subjected to a sham operation.

One week after surgery, the mice in the SS31 group were intraperitoneally injected with SS31 (3 mg/kg) once per day for 4 weeks. The mice in the NS group was intraperitoneally injected with the same dose of NS. The following groups were established, the right limb of the NS group was selected as sham + NS group (NS group-right limb), and the left limb of the NS group was selected as HLI + NS group (NS group-left limb). The right limb of the SS31 group was selected as sham + SS31 group (SS31 group-right limb) and the left limb of the SS31 group was selected as HLI + SS31 group (SS31 group-left limb). The mice were killed 24 h after the last dose of SS31 or NS was administered, and the gastrocnemius was collected. All procedures were authorized by the Animal Care Committee of Chongqing Medical University.

### Grip Strength

Grip strength of the mice was measured by the use of a grip force meter on the 7th, 14th, 21th, 28th, and 35th days after surgery. The mice were placed on the measuring device, the animals grasped the barbed wire tube with all limbs and pulled in parallel to the instrument until the mouse lost its grip. Three trials were performed with 1 min of rest between trials. The average of the three measurements was calculated as the grip strength of the mouse.

### Exhaustive Exercise

Exhaustive exercise was tested on the 7th, 14th, 21th, 28th, and 35th days after surgery to assess skeletal muscle function. At a speed of 8 m/min for 2 min, the speed of a running track increased by 2 m/s every 2 min until the mice could not run, or were driven back to the rest area within 2 s after being driven onto the track. The time and total distance were recorded.

### Gastrocnemius Index

The gastrocnemius index was calculated as the gastrocnemius weight (mg)/body weight (g).

### Hematoxylin and Eosin Staining

Gastrocnemius samples of each group were collected, fixed with 10% formaldehyde overnight, and then embedded in paraffin. The gastrocnemius were stained with hematoxylin and eosin (HE). The sections were observed using an inverted microscope. All images were observed and captured under × 400 magnification.

### Immunofluorescence

Tissue sections were fixed with 4% paraformaldehyde (PFA), permeabilized with 0.3% Triton X-100 for 30 min. Sections were washed three times with PBS, blocked with 10% normal goat serum and incubation with the following primary antibodies at 4°C overnight: anti-LC3II (Cell Signaling Technology, United States) and anti-LAMP1 (Santa Cruz Biotechnology, United States). The next day, they were incubated with Goat anti-Rabbit IgG (H + L) Cross-Adsorbed Secondary Antibody, Alexa Fluor 594 (Thermo Fisher, United States) and IFKine Green Donkey Anti-Mouse IgG (Abbkine, China) for 1 h at 37°C, and treated with DAPI for 10 min. The sections were viewed by an inverted microscope.

### Cell Culture and Establishment of the Hypoxia Model

C2C12 myoblasts were purchased from Zhongqiao Biotech and cultured in Dulbecco’s modified Eagle’s medium (DMEM) supplemented with 10% fetal bovine serum (FBS) and 1% penicillin/streptomycin in a constant-temperature incubator at 37°C with 5% CO_2_. Experiments were performed with 2% horse serum (HS), which induces cell differentiation and fusion.

CoCl_2_ is widely used throughout the world to simulate hypoxia. Ischemic myotubes were generated by incubating them in serum-free DMEM supplemented with 250 μM CoCl_2_ for 24 h.

### Cell Experimentation Groups

To investigate the relationship between SS31 (Qiang Yao Biological Company, China, with 99.14% purity) and autophagy in hypoxic myotubes. Rapamycin (RAPA) and chloroquine (CQ) were administered to stimulate and inhibit autophagy, respectively. Myotubes were randomly allocated into the following groups: the normoxic myotube group, hypoxic myotube group, hypoxic myotube + RAPA (15 nM) group, hypoxic myotube + SS31 (150 nM) + RAPA group, hypoxic myotube + CQ (8 μM) group, and hypoxic myotube + SS31 + CQ group.

### Transmission Electron Microscopy

After administration of the indicated treatment, myotubes were collected, loaded into centrifuge tubes and centrifuged at 1,500 rpm for 10 min, and then, the supernatant was aspirated. The cells and gastrocnemius samples were fixed with 2.5% glutaraldehyde for 18–20 h. After being rinsed, dehydrated and embedded, the samples were prepared under a dissecting microscope. Sections (1 μm) were prepared and then observed by transmission electron microscopy (TEM) (Hitachi-7700, Japan). Images were acquired, and five fields of view were randomly selected from each group for observation.

### Preparation of Mitochondria

Mitochondria were extracted from gastrocnemius muscle and myotubes according to the instructions of a tissue mitochondria isolation kit (Beyotime, C3606) and cell mitochondria isolation kit (Beyotime, C3601). The isolated mitochondria were added to mitochondrial isolation buffer for further detection.

### The Detection of Reactive Oxygen Species

The levels of ROS in the gastrocnemius and cells were measured using a ROS assay kit (Nanjing Jiancheng Biotech, China) according to the manufacturer’s instructions. After incubation with diluted dichlorodihydrofluorescein-diacetate (DCFH-DA) for 30 min, mitochondria of the gastrocnemius muscle and myotubes were detected with a Varioskan LUX Multimode Reader (Thermo Fisher, United States). BCA method was used to detect the concentration of isolated mitochondrial protein, which was used to standardize mitochondrial ROS. C2C12 cells were cultured in 6-well plates. Myotubes were resuspended in lysis buffer containing 10 μM DCFH-DA in serum-free medium for 30 min at 37°C and then washed twice with PBS. A fluorescence microscope (Olympus BX53, Olympus, Japan) was utilized to record the fluorescence intensity of intracellular ROS. ImageJ was used to estimate the average fluorescence intensity.

### Western Blot Assay

Tissue and cellular protein samples were prepared using RIPA lysis buffer. The following antibodies were used: anti-LC3 (Bimake, China), anti-SQSTM1/P62 (Proteintech, China), anti-Beclin1 (Proteintech, China), anti-ATG5 (Wanleibio, China), anti-Bax (Servicebio, China), anti-BCL2 (Proteintech, China), anti-cleaved caspase-3 (Proteintech, China), GAPDH (Proteintech, China), anti-mTOR (Cell Signaling Technology, United States), anti-mTOR Ser(P)2448 (Cell Signaling Technology, United States), anti-AKT (Cell Signaling Technology, United States), and anti-p-AKT (Cell Signaling Technology, United States). The total isolated protein was separated by SDS-polyacrylamide gel electrophoresis and transferred to polyvinylidene difluoride (PVDF) membranes (Millipore, United States), and the membranes were incubated overnight with a primary antibody at 4°C. The membranes were supplemented with secondary antibodies conjugated to horseradish peroxidase and subsequently detected with an enhanced chemiluminescent substrate (Millipore) and visualized *via* a Versa Doc imaging system (Bio-Rad, United States).

### Real-Time PCR

Total RNA from the gastrocnemius and myotube samples was extracted with TRIzol reagent (TaKaRa, Japan), and then, cDNA was obtained by reverse transcription using a PrimeScript RT reagent kit (TaKaRa, Japan). Then, cDNA was used to determine the mRNA expression levels. PCR was performed using SYBR Premix Ex Taq (TaKaRa, Japan) with SOD1-, SOD2-, CAT-, and GAPDH-specific primers. The following primer pairs were used: SOD1, 5′-CCAGTGCAGGACCTCATTTT-3′ (forward) and 5′-TTGTTTCTCATGGACCACCA-3′ (reverse); SOD2, 5′-CCGAGGAGAAGTACCACGAG-3′ (forward) and 5′-FCTTGATAGCCTCCAGCAAC-3′ (reverse); CAT, 5′-ACATGGTCTGGGACTTCTGG-3′ (forward) and 5′-CAAGT TTTTGATGCCCTGGT-3′ (reverse); and GAPDH, 5′-GGTT GTCTCCTGCGACTTCA-3′ (forward) and 5′-TGGTCCAGG GTTTCTTACTCC-3′ (reverse).

### Statistical Analysis

SPSS 25.0 and GraphPad Prism 8 were adopted to evaluate all data, which are presented as the means ± SD. Kolmogorov–Smirnov test was used to determine the normality of the distribution of continuous variables. One-way ANOVA and a two-sample *t*-test were performed to compare normally distributed variables. The Mann–Whitney *U* test was used for the comparison of skewed variables. The data are presented as the means ± SD. A *p*-value < 0.05 was considered statistically significant.

## Results

### SS31 Restored Skeletal Muscle Function in Hind Limb Ischemia Mice

**FIGURE 1:**
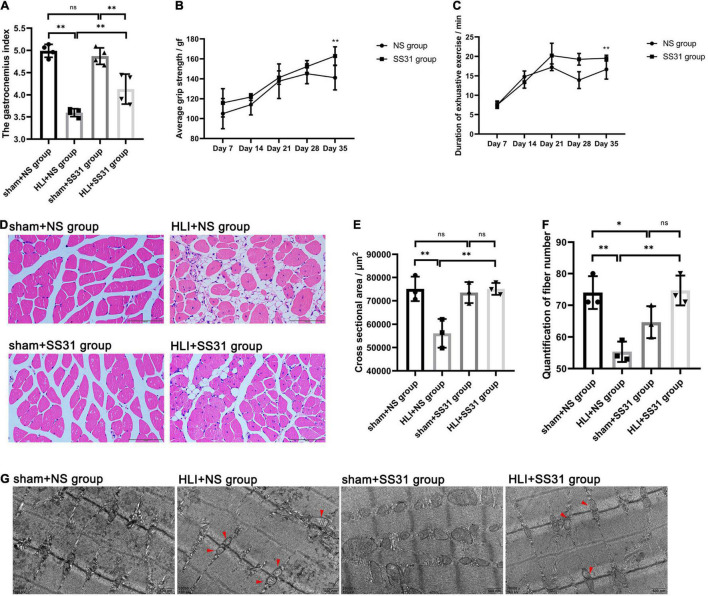
SS31 improves skeletal muscle function in HLI mouse model. **(A)** Gastrocnemius index. **(B)** Grip strength test. **(C)** Duration of exhaustive exercise in minutes. **(D–F)** Skeletal muscle of the four groups was stained with HE (magnification of ×400). ImageJ was used to measure the fiber number and cross-sectional area of the gastrocnemius fibers. **(G)** TEM indicated that mitochondria were swollen and the Z-lines disorganized in HLI mice; however, SS31 treatment partially reversed these changes (red arrowheads indicate damaged mitochondria) (magnification of ×30,000). *n* = 3–6. *p < 0.05, ^**^p < 0.01.

To determine whether SS31 affects the muscle function of mice with HLI, skeletal muscle function was examined after ischemic injury with or without SS-31 administration. The gastrocnemius index decreased after HLI was induced in mice (Figure 1A), but SS31 treatment gradually led to recovery of the gastrocnemius index of the ischemic limb. In the grip strength test, the grip strengths of the NS group mice were significantly lower than those of the SS31 group mice on day 35 (Figure 1B). Moreover, the duration of exhaustive exercise was substantially reduced in the NS group at day 35, compared to that of the SS31 group (Figure 1C), which was consistent with the results of the grip strength test. These results suggested that SS31 administration can enhance the recovery of strength and function of ischemic limbs.

### SS31 Alleviated Injury of Ischemic Skeletal Muscle

Hematoxylin and eosin staining showed that skeletal muscle tissue of the HLI + NS group mice showed vast adipocyte infiltration, high levels of interstitial edema, smaller cross-sectional areas, and wider intermuscular spaces (Figures 1D–F). However, SS31 markedly reversed these changes. The fiber numbers decreased after HLI, but SS31 treatment gradually led to the recovery of fiber numbers in mice with HLI. We counted the number of fibers with ImageJ software and found that SS31 treatment significantly increased the number of ischemic skeletal muscle fibers.

Furthermore, with TEM, we found mitochondrial swells and Z-line disorders in the skeletal muscle after HLI injury (Figure 1G), but this damage was markedly reduced after SS31 treatment.

### SS31 Reversed the Promotion of Apoptosis and Impairment of Autophagic Flux Induced by Hind Limb Ischemia

**FIGURE 2:**
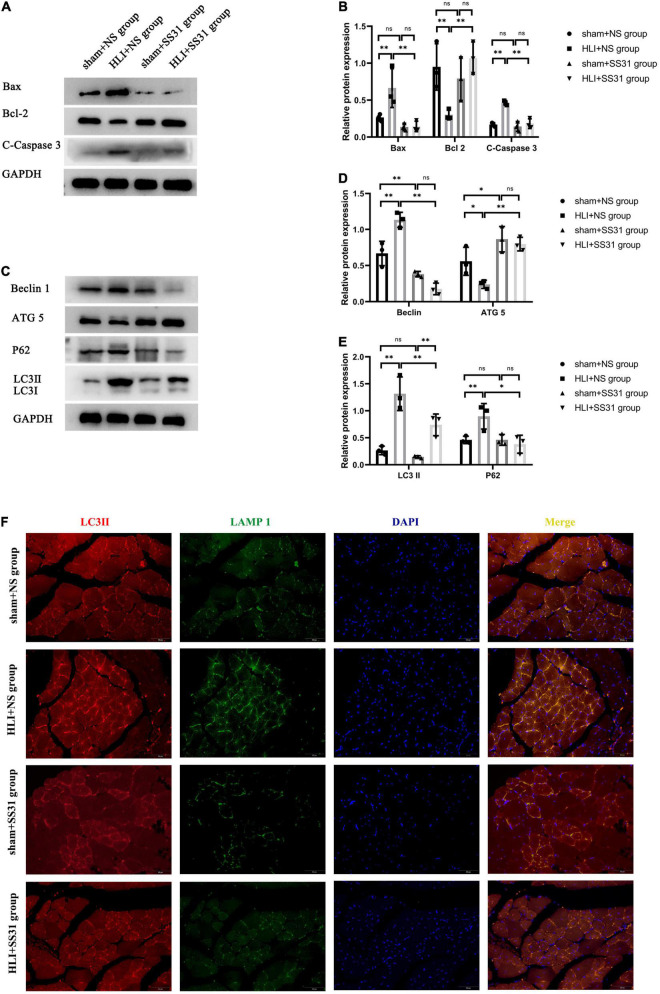
SS31 inhibits apoptosis and restores autophagic flux in HLI mouse model. **(A,B)** The expression levels of apoptosis-related proteins were detected by WB. GAPDH was used as a control. **(C–E)** Immunoblot analysis showing the protein levels of LC3 II, P62, Beclin1, and ATG5. Western blot results were analyzed for LC3 II expression and normalized to GAPDH expression, P62 normalized to GAPDH, Beclin1 normalized to GAPDH, and ATG5 normalized to GAPDH. **(F)** LC3II (red) and LAMP1 (green) were detected by immunofluorescence. Nuclei are labeled with DAPI (blue) (magnification of ×400). *n* = 3. *p < 0.05, ^**^p < 0.01.

After HLI, skeletal muscle tissues show an increased apoptosis rate. The results of apoptosis-related protein expression demonstrated that the expression of cleaved caspase3 (C-caspase3) and Bax (proapoptotic proteins) in skeletal muscle tissue in the HLI + NS group was upregulated compared with that in the sham + NS group, with the most profound difference in C-caspase3 in skeletal muscle. The expression of pro-apoptotic proteins was reduced in the sham + SS31 group compared to the sham + NS group, but the difference did not reach the significance (Figures 2A,B). The level of Bcl-2 (an antiapoptotic protein) was remarkably reduced in the HLI + NS group. SS31 greatly reduced the cell apoptosis rate that had been increased by HLI. These findings indicated that the SS31 peptide protects skeletal muscle from apoptosis.

To investigate the effects of SS31 on autophagy in mice with HLI, we evaluated the expression levels of autophagy-related markers LC3 II, P62, Beclin1, and ATG5 by Western blot analysis (Figures 2C–E). Compared to the sham + NS group, there was no significant decrease in the levels of LC3II, P62 in the sham + SS31 group, while the level of Beclin1 was significantly reduced in the sham + SS31 group. The data indicated that the protein expression levels of LC3II, P62, and Beclin1 were significantly increased in HLI mice, but SS31 treatment remarkably decreased the levels of these autophagy-related markers. Furthermore, the expression level of ATG5 in SS31-treated HLI mice was significantly higher than that in HLI mice without SS31 treatment. In comparison with the sham + NS group, the expression level of ATG5 was statistically elevated in the sham + SS31 group. Double immunofluorescence (IF) staining analysis was used to detect the LC3II and the lysosomal-associated membrane protein 1 (LAMP-1) (Figure 2F). We observed the colocalization of LC3II and LAMP1 was increased in HLI mice. However, colocalization of LC3II and LAMP1 was reduced in hindlimb ischemic mice after SS31 intervention. These data indicated that SS31 can restore impaired autophagic flux in HLI mice.

### SS31 Attenuated Oxidative Stress in the Hind Limbs of Ischemic Mice

**FIGURE 3:**
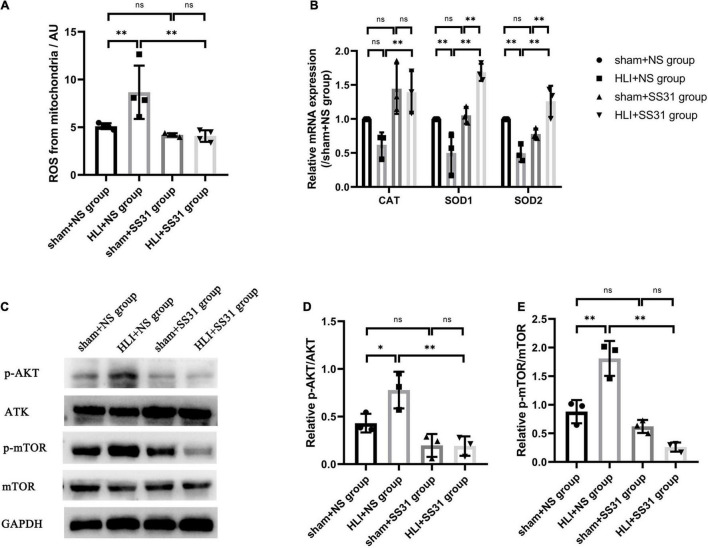
SS31 alleviates oxidative stress and inhibits AKT-mTOR pathway in HLI mouse model. **(A)** A reactive oxygen species assay kit was used to quantify mitochondrial ROS production. **(B)** The relative mRNA levels of SOD1, SOD2, and CAT were determined by RT-qPCR. **(C–E)** The protein levels of phosphorylated AKT and mTOR in HLI mice were determined. The data were standardized on the basis of the GAPDH level, *n* = 3–4. *p < 0.05, ^**^p < 0.01.

We isolated mitochondria from skeletal muscle at first, and then tested the level of mitochondrial ROS in this study. mitochondrial ROS production was considerably increased in the HLI + NS group compared to the sham + NS group (Figure 3A). SS31 significantly decreased mitochondrial ROS levels in mice with HLI compared with those in the HLI + NS group. However, there were no significant differences in the content of mitochondrial ROS between the sham + SS31 group and the sham + NS group. Furthermore, to validate the effect of SS31 on oxidative stress in the skeletal muscle of HLI mice, we measured the mRNA of the three antioxidant enzymes (SOD1, SOD2, and CAT) (Figure 3B). Compared with that of the sham + NS group, the mRNA expression of SOD1 and SOD2 was remarkably decreased in the HLI + NS group, and the mRNA expression of CAT in the HLI + NS group was less than that in the sham + NS group, although the difference did not reach statistical significance. Compared with that in the HLI + NS group, the mRNA levels of SOD1, SOD2, and CAT in the HLI + SS31 group were significantly upregulated. Compared with the sham + NS group, the mRNA levels of CAT and SOD1 were enhanced in the sham + SS31 group, their differences did not reach statistical significance, while the mRNA level of SOD2 was slightly reduced. The results suggest that SS31 can alleviate oxidative damage caused by ischemia by promoting the expression of antioxidant enzymes.

### SS31 Inhibited the AKT-mTOR Pathway in Hind Limb Ischemia Mice

To assess the impact of SS31 on the AKT-mTOR pathway in HLI mice. Western blotting was performed to detect the expression of phosphorylated AKT (p-AKT) and phosphorylated mTOR (p-mTOR) in HLI mice (Figures 3C–E). The immunoblot analysis indicated that the expression levels of p-AKT and p-mTOR were higher in the HLI group than in the control group. These levels decreased significantly after SS31 treatment. As compared to the sham + NS group, the levels of p-AKT and p-mTOR were slightly decreased in the sham + SS31 group.

### SS31 Improved Hypoxia-Induced Blockage of Autophagic Flux in C2C12 Myotubes

**FIGURE 4:**
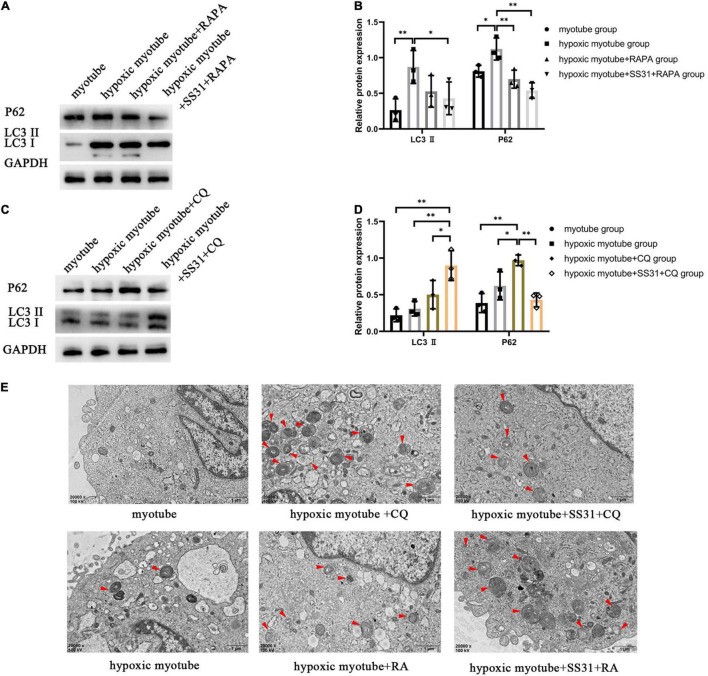
SS31 activates autophagic flux in C2C12 myotubes. **(A,B)** RAPA was the intervention for hypoxic myotubes treated with or without SS31. The expression of autophagy-related proteins LC3 II and P62 were examined. **(C,D)** CQ was the intervention for hypoxic myotubes with or without SS31 treatment, and in the lysates, LC3 II and P62 expression was detected by immunoblotting. **(E)** Autophagy vacuoles were observed *via* electron microscopy (EM) (magnification of ×20,000). Red arrowheads represent autophagosomes. The data were standardized on the basis of the GAPDH level. *n* = 3. *p < 0.05, ^**^p < 0.01.

To explore the autophagy process induced by SS31, we treated hypoxic myotubes with CQ, RAPA, and SS31. Compared with those in the myotube group, a higher LC3 II ratio and a higher level of P62 were found in cells under hypoxic conditions. Intracellular autophagy-related marker levels with or without SS31 treatment were detected. Hypoxia-induced changes in LC3 II and P62 expression were reversed by the autophagy activator RAPA (Figures 4A,B). This situation was more obvious after the SS31 intervention. CQ greatly exacerbated the increase in hypoxia-induced LC3 II and P62 expression. The LC3 II level increased after SS31 treatment. In contrast, the level of P62 was decreased (Figures 4C,D).

As observed through TEM, after hypoxia, the ultrastructure of myotubes was injured; for example, the outer mitochondrial compartment was swollen, the endoplasmic reticulum was dilated, and autophagosomes were found in the cytoplasm (Figure 4E). In the hypoxic myotube + RAPA + SS31 group, the number of autolysosomes was further increased, the damage was less extensive than in the hypoxic myotube + RAPA group. Compared with the hypoxic myotube + CQ group, the hypoxic myotube + CQ + SS31 group exhibited a decrease in the number of autophagosomes and a decrease in the number of damaged mitochondria. These results indicated that SS31 improves autophagic flux and promotes autophagosome clearance in myotubes following hypoxic injury.

### SS31 Alleviated Hypoxia-Induced Oxidative Stress in C2C12 Myotubes

**FIGURE 5:**
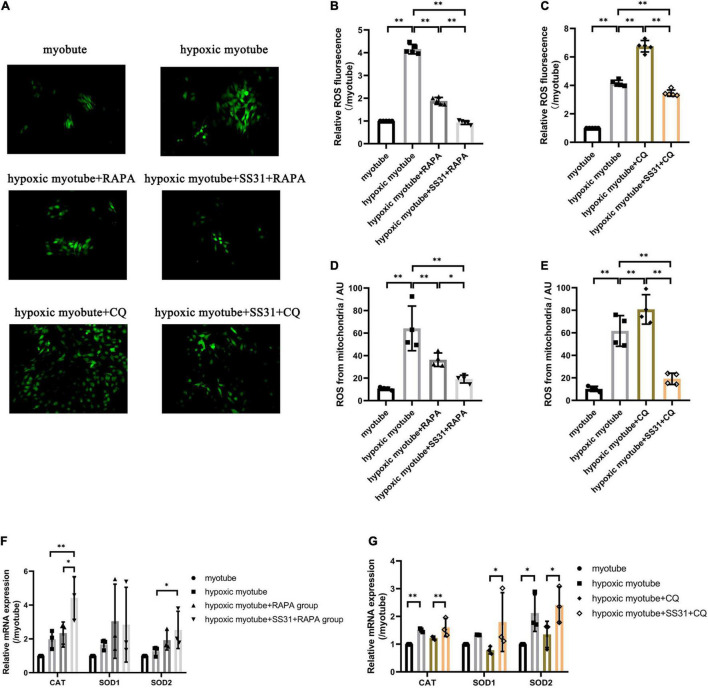
SS31 attenuates oxidative stress in C2C12 myotubes. **(A–C)** ROS contents in C2C12 myotubes. **(D,E)** DCFH-DA staining analysis revealed the mitochondrial ROS levels. **(F,G)** The relative mRNA expression levels of SOD1, SOD2, and CAT were detected by RT-qPCR. The data were standardized on the basis of the GAPDH level, *n* = 3–5. *p < 0.05, ^**^p < 0.01.

Evidence has suggested that a regulatory interaction exists between autophagy and oxidative stress (Figures 5A–G). To elucidate the relationship between autophagy and oxidative stress in PAD, we measured the ROS content and mRNA expression levels of three antioxidant enzymes. In contrast to its effect on hypoxic myotubes, CQ markedly increased ROS levels and decreased the expression levels of SOD1, SOD2, and CAT. Compared with the hypoxic myotube + CQ group, in the hypoxic myotube + SS31 + CQ group, ROS contents were reduced, and the levels of the three antioxidant enzymes SOD1, SOD2, and CAT were significantly increased. Compared with its effect on hypoxic myotubes, RAPA increased the levels of the three antioxidant enzymes, but the difference did not reach statistical significance, and a decrease in ROS levels was observed in the hypoxic myotube + RAPA group. Compared with those in the hypoxic myotube + RAPA group, the ROS levels decreased and the mRNA levels of SOD2 and CAT were increased more significantly in the hypoxic myotube + SS31 + RAPA group. Interestingly, the mRNA expression level of SOD1 was slightly downregulated in the hypoxic myotube + SS31 + RAPA group.

### SS31 Attenuated AKT-mTOR Signaling in C2C12 Myotubes

**FIGURE 6:**
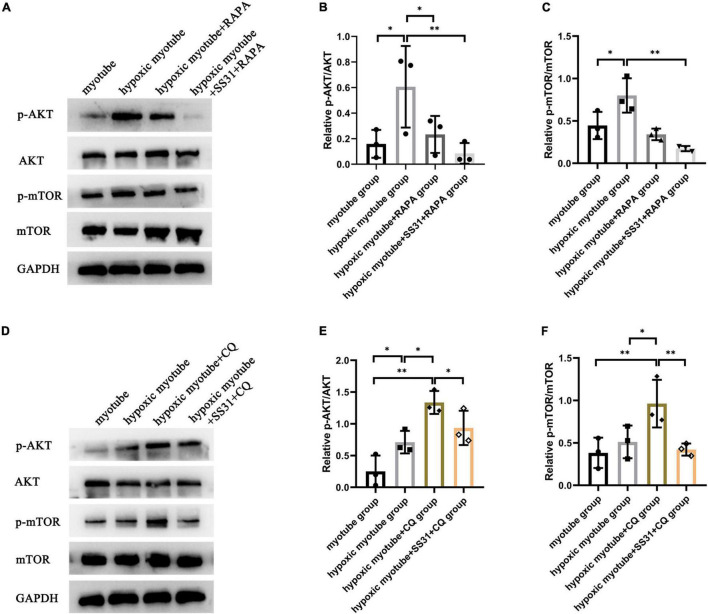
SS31 suppresses AKT-mTOR pathway in C2C12 myotubes. **(A–C)** RAPA was used as the intervention in hypoxic myotubes in the presence or absence of SS31, and the levels of p-AKT and p-mTOR in C2C12 myotubes were detected. **(D–F)** CQ was used as the intervention in hypoxic myotubes with or without SS31 treatment. The expression levels of p-AKT and p-mTOR were examined, *n* = 3. *p < 0.05, ^**^p < 0.01.

To further understand whether the AKT-mTOR signaling pathway is involved in SS31-induced autophagy in hypoxic myotubes, we treated hypoxic myotubes with SS31, CQ, and RAPA and detected the expression levels of p-AKT and p-mTOR by Western blotting (Figures 6A–F). The results demonstrated that the levels of p-AKT and p-mTOR in hypoxic myotubes increased significantly compared with that in the myotube group. After CQ intervention, the levels of p-AKT and p-mTOR increased further, and SS31 intervention reversed this change. In contrast, the levels of p-AKT and p-mTOR decreased significantly after RAPA treatment, an outcome exacerbated by SS31 intervention. These results indicated that the AKT-mTOR signaling pathway is likely engaged in SS31-mediated autophagy regulation.

## Discussion

Peripheral artery disease is often accompanied by skeletal muscle mitochondrial dysfunction and oxidative stress (25). SS31 has the advantages of a small molecular weight, stable structure, fast absorption, weak toxicity, and long half-life (19); therefore, it is the most recognized small-molecule peptide among many mitochondrial targeted antioxidants. During all the experiments, no adverse drug reactions were evident in the mice. A previous study has shown that SS31 improves skeletal muscle function by reversing mitochondrial damage and reducing mitochondrial oxidant production in the context of sarcopenia (26). However, the application of SS31 to treat PAD is still very rare. In this study, we demonstrated, for the first time, that SS31 can improve oxidative stress by restoring autophagic flux to protect the skeletal muscle of the hind limbs of ischemic mice, while based on our findings, the basal effect of SS31 seemed to be small.

Our study revealed that the skeletal muscle function of NS-treated mice and SS31-treated mice recovered starting 21 days after femoral artery ligation. Although C57BL/6 mice show a certain degree of resistance to ischemia, a previous study has shown that an HLI model established with old mice is stable. Persistent chronic hypoperfusion occurs several weeks after arterial ligation of these mice (9). HLI induction resulted in the loss of muscle strength and muscle mass. The SS31 intervention remarkably accelerated the recovery of skeletal muscle function and mass that had been lost because of ischemia.

The ischemic limb presents a reduced calf skeletal muscle area and increased calf muscle fat cell infiltration and greater fibrosis in patients with PAD (27). These pathophysiological outcomes also appeared in the HLI mouse models in this study. SS31 administration markedly reversed these changes. Cell apoptosis was inhibited and cell proliferation was increased by SS31 intervention. Calf muscle biopsy samples of patients with PAD, chronic ischemic skeletal muscle is often characterized by mitochondrial dysfunction (28), and this dysfunction was found in our research.

Autophagic flux is a dynamic process of degrading and recycling autophagosomes. Chenshu found that restoration of autophagic flux by RAPA intervention during ischemia can alleviate skeletal muscle injury, indicating that promoting autophagy has a potentially protective effect on IR in mice (29). In a study by In-Hye Jeong, ischemia induced autophagy in endothelial cells, and this increase in autophagy contributed to the regeneration and recovery of injured skeletal muscle (30). Wensi found that endothelial mTORC1 deletion can activate autophagy to protect against HLI injury in diabetic mice (31). Chronic limb ischemia results in progressive and severe damage to skeletal muscle. Research has shown that insufficient autophagic flux may be harmful to muscles, inducing conditions such as colchicine myopathy and Danon disease (32). In the present study, we also observed insufficient autophagic flux in the HLI mice, but SS31 treatment reversed these changes. To further study the relationship between SS31 and autophagic flux, an autophagy inhibitor (CQ) and inducer (RAPA) were used for *in vitro* experiments. The results of the *in vitro* experiments supported the aforementioned results. mTOR is activated by AKT, resulting in increased skeletal muscle protein synthesis (33). The veracity of this supposition is supported by genetic evidence. Knocking out mTOR has been shown to lead to progressive myopathy because skeletal muscle mass and function are affected (34). These data indicated that SS31 administration contributes to the recovery of autophagic flux *via* the AKT-mTOR signaling pathway in hindlimb ischemic mice.

A recent study showed that oxidative stress was increased in a model of critical limb ischemia (35). Mitochondrial ROS play critical roles in the pathogenesis of PAD (6). This phenomenon was observed in our experiments in this study. Previous studies have shown that combined SS31-Mito therapy protected mitochondrial integrity by suppressing oxidative stress and inflammation after IR injury (36). SC-TK-SS31 intervention (SS31 prepared by binding to l-serine–modified chitosan through a ROS-sensitive thioketal junction) reduced inflammation, apoptosis and oxidative stress to protect mitochondria in AKI (37). SS31, as a cellular antioxidant peptide, can reduce oxidative stress to protect cells from hypoxic injury. Autophagy contributes to the decrease in oxidative stress in skeletal muscle. Many studies have shown that impairment of autophagy leads to the cytoplasmic accumulation of ubiquitinated proteins, resulting in mitochondrial damage and promoting the production of ROS (38). Although the initial target of SS31 is mitochondria, it has also been shown that SS31 can act directly on autophagy (39, 40). We explored the relationship between SS31, autophagy and oxidative stress. Hypoxia led to profound increases in ROS levels. CQ increased total and mitochondrial ROS levels in hypoxic myotubes, and SS31 counteracted these ROS level changes to some extent and upregulated three antioxidant enzymes. In addition, RAPA decreased the content of total and mitochondrial ROS in hypoxic myotubes, and SS31 further reduced these ROS levels and significantly increased the mRNA level of CAT. These results indicated that SS31 can reduce oxidative stress by promoting autophagy. Interestingly, compared with the effect in the myotube group, hypoxia increased the levels of three antioxidant enzymes in myotubes, which may have been a result of our cellular model not fully mimicking the pathophysiological state of HLI mice.

Our study has advantages. First, our findings revealed that SS31 can reduce oxidative stress by restoring autophagic flux to improve skeletal muscle structure and function, and SS31 may be a new treatment strategy for PAD. Second, we demonstrated, for the first time, that SS31 may improves the structure and function of skeletal muscle in hindlimb ischemic mice through the AKT-mTOR signaling pathway.

## Conclusion

In conclusion, SS31 could restore skeletal muscle function, ameliorate skeletal muscle injury, and decrease the apoptosis rate in hindlimb ischemic mice. Therefore, we hypothesized that SS31 could improve the symptoms of elderly PAD patients. Moreover, SS31 can inhibit oxidative stress by restoring autophagic flux and thus reverse hypoxia-induced injury, which may be achieved through the AKT-mTOR pathway. This study indicated that SS31 can be used as a potential PAD medicine.

## Data Availability Statement

The original contributions presented in the study are included in the article/Supplementary Material, further inquiries can be directed to the corresponding authors.

## Ethics Statement

The animal study was reviewed and approved by the Animal Care Committee of Chongqing Medical University.

## Author Contributions

QY and CL contributed to the conception, study design, experimental implementation, data analysis, and manuscript writing. QC was involved in article conception, supervision, and project management. All authors contributed to the article and approved the submitted version.

## Conflict of Interest

The authors declare that the research was conducted in the absence of any commercial or financial relationships that could be construed as a potential conflict of interest.

## Publisher’s Note

All claims expressed in this article are solely those of the authors and do not necessarily represent those of their affiliated organizations, or those of the publisher, the editors and the reviewers. Any product that may be evaluated in this article, or claim that may be made by its manufacturer, is not guaranteed or endorsed by the publisher.

## References

[1] Cooke JP Chen Z. A compendium on peripheral arterial disease. *Circ Res.* (2015) 116:1505–8. 10.1161/circresaha.115.306403 25908724 PMC4410267

[2] Leeper NJ Kullo IJ Cooke JP. Genetics of peripheral artery disease. *Circulation.* (2012) 125:3220–8. 10.1161/circulationaha.111.033878 22733336 PMC3755373

[3] Teraa M Conte MS Moll FL Verhaar MC. Critical limb ischemia: current trends and future directions. *J Am Heart Assoc.* (2016) 5:e002938. 10.1161/jaha.115.002938 26908409 PMC4802465

[4] Ismaeel A Brumberg RS Kirk JS Papoutsi E Farmer PJ Bohannon WT Oxidative stress and arterial dysfunction in peripheral artery disease. *Antioxidants.* (2018) 7:145. 10.3390/antiox7100145 30347720 PMC6210426

[5] Pipinos II Judge AR Selsby JT Zhu Z Swanson SA Nella AA The myopathy of peripheral arterial occlusive disease: part 1. Functional and histomorphological changes and evidence for mitochondrial dysfunction. *Vasc Endovasc Surg.* (2007) 41:481–9. 10.1177/1538574407311106 18166628

[6] Steven S Daiber A Dopheide JF Münzel T Espinola-Klein C. Peripheral artery disease, redox signaling, oxidative stress – basic and clinical aspects. *Redox Biol.* (2017) 12:787–97. 10.1016/j.redox.2017.04.017 28437655 PMC5403804

[7] Signorelli SS Scuto S Marino E Xourafa A Gaudio A. Oxidative stress in peripheral arterial disease (PAD) mechanism and biomarkers. *Antioxidants (Basel).* (2019) 8:367. 10.3390/antiox8090367 31480714 PMC6770183

[8] Signorelli SS Vanella L Abraham NG Scuto S Marino E Rocic P. Pathophysiology of chronic peripheral ischemia: new perspectives. *Ther Adv Chronic Dis.* (2020) 11:2040622319894466. 10.1177/2040622319894466 32076496 PMC7003198

[9] He W Wang P Chen Q Li C. Exercise enhances mitochondrial fission and mitophagy to improve myopathy following critical limb ischemia in elderly mice via the PGC1a/FNDC5/irisin pathway. *Skelet Muscle.* (2020) 10:25. 10.1186/s13395-020-00245-2 32933582 PMC7490877

[10] Gerhard-Herman MD Gornik HL Barrett C Barshes NR Corriere MA Drachman DE 2016 AHA/ACC guideline on the management of patients with lower extremity peripheral artery disease: a report of the American college of cardiology/American heart association task force on clinical practice guidelines. *Circulation.* (2017) 135:e726–79. 10.1161/cir.0000000000000471 27840333 PMC5477786

[11] Fowkes FG Rudan D Rudan I Aboyans V Denenberg JO McDermott MM Comparison of global estimates of prevalence and risk factors for peripheral artery disease in 2000 and 2010: a systematic review and analysis. *Lancet.* (2013) 382:1329–40. 10.1016/s0140-6736(13)61249-0 23915883

[12] Parzych KR Klionsky DJ. An overview of autophagy: morphology, mechanism, and regulation. *Antioxid Redox Signaling.* (2014) 20:460–73. 10.1089/ars.2013.5371 23725295 PMC3894687

[13] Cheng Y Ren X Hait WN Yang JM. Therapeutic targeting of autophagy in disease: biology and pharmacology. *Pharmacol Rev.* (2013) 65:1162–97. 10.1124/pr.112.007120 23943849 PMC3799234

[14] Liang D Han D Fan W Zhang R Qiao H Fan M Therapeutic efficacy of apelin on transplanted mesenchymal stem cells in hindlimb ischemic mice via regulation of autophagy. *Sci Rep.* (2016) 6:21914. 10.1038/srep21914 26902855 PMC4763210

[15] Schiattarella GG Hill JA. Therapeutic targeting of autophagy in cardiovascular disease. *J Mol Cell Cardiol.* (2016) 95:86–93. 10.1016/j.yjmcc.2015.11.019 26602750 PMC4871782

[16] McClung JM McCord TJ Ryan TE Schmidt CA Green TD Southerland KW BAG3 (Bcl-2-associated athanogene-3) coding variant in mice determines susceptibility to ischemic limb muscle myopathy by directing autophagy. *Circulation.* (2017) 136:281–96. 10.1161/circulationaha.116.024873 28442482 PMC5537727

[17] Birk AV Liu S Soong Y Mills W Singh P Warren JD The mitochondrial-targeted compound SS-31 re-energizes ischemic mitochondria by interacting with cardiolipin. *J Am Soc Nephrol.* (2013) 24:1250–61. 10.1681/asn.2012121216 23813215 PMC3736700

[18] Dai DF Chen T Szeto H Nieves-Cintrón M Kutyavin V Santana LF Mitochondrial targeted antioxidant peptide ameliorates hypertensive cardiomyopathy. *J Am Coll Cardiol.* (2011) 58:73–82. 10.1016/j.jacc.2010.12.044 21620606 PMC3742010

[19] Szeto HH. Mitochondria-targeted cytoprotective peptides for ischemia-reperfusion injury. *Antioxid Redox Signal.* (2008) 10:601–19. 10.1089/ars.2007.1892 17999629

[20] Zhao K Zhao GM Wu D Soong Y Birk AV Schiller PW Cell-permeable peptide antioxidants targeted to inner mitochondrial membrane inhibit mitochondrial swelling, oxidative cell death, and reperfusion injury. *J Biol Chem.* (2004) 279:34682–90. 10.1074/jbc.M402999200 15178689

[21] Cho S Szeto HH Kim E Kim H Tolhurst AT Pinto JT. A novel cell-permeable antioxidant peptide, SS31, attenuates ischemic brain injury by down-regulating CD36. *J Biol Chem.* (2007) 282:4634–42. 10.1074/jbc.M609388200 17178711

[22] Wyss JC Kumar R Mikulic J Schneider M Mary JL Aebi JD Differential effects of the mitochondria-active tetrapeptide SS-31 (D-Arg-dimethylTyr-Lys-Phe-NH) and its peptidase-targeted prodrugs in experimental acute kidney injury. *Front Pharmacol.* (2019) 10:1209. 10.3389/fphar.2019.01209 31780923 PMC6857474

[23] Thomas DA Stauffer C Zhao K Yang H Sharma VK Szeto HH Mitochondrial targeting with antioxidant peptide SS-31 prevents mitochondrial depolarization, reduces islet cell apoptosis, increases islet cell yield, and improves posttransplantation function. *J Am Soc Nephrol.* (2007) 18:213–22. 10.1681/asn.2006080825 17151329

[24] Zhang CX Cheng Y Liu DZ Liu M Cui H Zhang BL Mitochondria-targeted cyclosporin A delivery system to treat myocardial ischemia reperfusion injury of rats. *J Nanobiotechnol.* (2019) 17:18. 10.1186/s12951-019-0451-9 30683110 PMC6346555

[25] Paradis S Charles AL Georg I Goupilleau F Meyer A Kindo M Aging exacerbates ischemia-reperfusion-induced mitochondrial respiration impairment in skeletal muscle. *Antioxidants (Basel).* (2019) 8:168. 10.3390/antiox8060168 31181751 PMC6616544

[26] Campbell MD Duan J Samuelson AT Gaffrey MJ Merrihew GE Egertson JD Improving mitochondrial function with SS-31 reverses age-related redox stress and improves exercise tolerance in aged mice. *Free Radic Biol Med.* (2019) 134:268–81. 10.1016/j.freeradbiomed.2018.12.031 30597195 PMC6588449

[27] McDermott MM Ferrucci L Gonzalez-Freire M Kosmac K Leeuwenburgh C Peterson CA Skeletal muscle pathology in peripheral artery disease: a brief review. *Arterioscler Thromb Vasc Biol.* (2020) 40:2577–85. 10.1161/atvbaha.120.313831 32938218 PMC9571495

[28] Gratl A Pesta D Gruber L Speichinger F Raude B Omran S The effect of revascularization on recovery of mitochondrial respiration in peripheral artery disease: a case control study. *J Transl Med.* (2021) 19:244. 10.1186/s12967-021-02908-0 34088309 PMC8178834

[29] Liu C Peng M Zheng L Zhao Y Wang R Su Q Enhanced autophagy alleviates injury during hindlimb ischemia/reperfusion in mice. *Exp Ther Med.* (2019) 18:1669–76. 10.3892/etm.2019.7743 31410124 PMC6676216

[30] Jeong IH Bae WY Choi JS Jeong JW. Ischemia induces autophagy of endothelial cells and stimulates angiogenic effects in a hindlimb ischemia mouse model. *Cell Death Dis.* (2020) 11:624. 10.1038/s41419-020-02849-4 32796816 PMC7429831

[31] Fan W Han D Sun Z Ma S Gao L Chen J Endothelial deletion of mTORC1 protects against hindlimb ischemia in diabetic mice via activation of autophagy, attenuation of oxidative stress and alleviation of inflammation. *Free Radic Biol Med.* (2017) 108:725–40. 10.1016/j.freeradbiomed.2017.05.001 28473248

[32] Margeta M. Autophagy defects in skeletal myopathies. *Annu Rev Pathol.* (2020) 15:261–85. 10.1146/annurev-pathmechdis-012419-032618 31594457

[33] Schiaffino S Dyar KA Ciciliot S Blaauw B Sandri M. Mechanisms regulating skeletal muscle growth and atrophy. *FEBS J.* (2013) 280:4294–314. 10.1111/febs.12253 23517348

[34] Risson V Mazelin L Roceri M Sanchez H Moncollin V Corneloup C Muscle inactivation of mTOR causes metabolic and dystrophin defects leading to severe myopathy. *J Cell Biol.* (2009) 187:859–74. 10.1083/jcb.200903131 20008564 PMC2806319

[35] Lejay A Choquet P Thaveau F Singh F Schlagowski A Charles AL A new murine model of sustainable and durable chronic critical limb ischemia fairly mimicking human pathology. *Eur J Vasc Endovasc Surg.* (2015) 49:205–12. 10.1016/j.ejvs.2014.12.010 25579876

[36] Lee FY Shao PL Wallace CG Chua S Sung PH Ko SF Combined therapy with SS31 and mitochondria mitigates myocardial ischemia-reperfusion injury in rats. *Int J Mol Sci.* (2018) 19:2782. 10.3390/ijms19092782 30223594 PMC6164143

[37] Liu D Shu G Jin F Qi J Xu X Du Y ROS-responsive chitosan-SS31 prodrug for AKI therapy via rapid distribution in the kidney and long-term retention in the renal tubule. *Sci Adv.* (2020) 6:eabb7422. 10.1126/sciadv.abb7422 33036968 PMC7546709

[38] Rodney GG Pal R Abo-Zahrah R. Redox regulation of autophagy in skeletal muscle. *Free Radic Biol Med.* (2016) 98:103–12. 10.1016/j.freeradbiomed.2016.05.010 27184957 PMC4975974

[39] Smuder AJ Sollanek KJ Nelson WB Min K Talbert EE Kavazis AN Crosstalk between autophagy and oxidative stress regulates proteolysis in the diaphragm during mechanical ventilation. *Free Radic Biol Med.* (2018) 115:179–90. 10.1016/j.freeradbiomed.2017.11.025 29197632 PMC5767544

[40] Montalvo RN Doerr V Min K Szeto HH Smuder AJ. Doxorubicin-induced oxidative stress differentially regulates proteolytic signaling in cardiac and skeletal muscle. *Am J Physiol Regul Integr Comp Physiol.* (2020) 318:R227–33. 10.1152/ajpregu.00299.2019 31774307

