# Supplementary material for: SS31 Ameliorates Oxidative Stress via the Restoration of Autophagic Flux to Protect Aged Mice From Hind Limb Ischemia
Source: Front Cardiovasc Med. 2022 Apr 14;9:789331. doi: 10.3389/fcvm.2022.789331 (PMC9046554; doi:10.3389/fcvm.2022.789331)
Supplement: Supplementary file 1 [file Data_Sheet_1.docx]

**the download links ：**

<https://www.jianguoyun.com/p/DbiztYQQyLbzCRjZ9q8E>
